# Editorial: Innovative approaches to cholangiocarcinoma: diagnosis, treatment, and multidisciplinary care

**DOI:** 10.3389/fonc.2025.1717296

**Published:** 2025-12-02

**Authors:** Karol Rawicz-Pruszyński, Piero Boraschi, Lorenzo Fornaro

**Affiliations:** 1 Department of Surgical Oncology, Medical University of Lublin, Lublin, Poland; 2 Department of Diagnostic, Interventional Radiology and Nuclear Medicine, Azienda Ospedaliero-Universitaria Pisana, Pisa, Italy; 3 Department of Oncology, Azienda Ospedaliero-Universitaria Pisana, Pisa, Italy

**Keywords:** cholangiocacinoma, biliary cancer, diagnosis, treatment, multidisciplinary approach

Biliary tract cancer (BTC) is a heterogenous group of malignancies, comprising intrahepatic, extrahepatic and perihilar cholangiocarcinoma, as well as gallbladder cancer. BTC is generally recognized as an aggressive disease, surgery representing the only potentially curative treatment. However, recurrence rates after resection remain high, with an even larger proportion of patients still presenting with unresectable or overtly metastatic disease at diagnosis (1). In such cases, palliative systemic therapy is the mainstay of treatment (2). Challenging issues in BTC management can be faced by clinicians during the entire course of the disease, from radiological and histopathological diagnosis, through molecular characterization, to treatment of the disease and palliation of symptoms. In recent years, several advances in the approach to such a difficult clinical scenario has been achieved (1, 2). In this Research Topic we aim to describe some of the most recent improvements in the diagnosis, prognostic classification and treatment of BTC.

In particular, Liu et al. and Lang et al. provided intriguing insights into the identification of prognostic indicators in BTC. In their works, several routinely available parameters (correlated with the inflammatory, nutritional, and immune status of patients) have been either confirmed or identified as valuable markers, to stratify the whole patient population into different prognostic subgroups in different disease settings, beyond the conventional classification based on the site of tumor origin along the biliary tree. This could be of particular interest for clinicians, in order to personalize treatment approaches as well as follow up strategies, and could be implemented into future studies as stratification factors to properly assess treatment impact into different patient subsets (3). Moreover, molecular characterization of BTC is gaining momentum in the oncology community, but the prognostic role of major biological alterations identified in BTC is still under investigation (4): the ability to provide prognostic information by easily accessible parameters (such those described in the included articles) is therefore of relevance to ensure the best prognostic classification in different clinical situations.

Other authors contributed with systematic review or original researches to the current debate about the role of immune checkpoint inhibitors (ICIs) and tyrosine kinase inhibitors (TKIs) in BTC management. There is no doubt that the introduction of ICIs, such as durvalumab and pembrolizumab, into the treatment armamentarium against BTC has represented a major advancement in this disease (5). However, optimization of ICI treatment is still far from its final goal, and head-to-head comparison between different agents has not been reported. Currently, the choice between different agents is mainly based on indirect comparisons of safety and efficacy data from trials with different designs and patient populations, and in this regard the attempt to provide a more detailed analysis is therefore of value.

Finally, development of chemotherapy-free strategies based on the association of ICIs and TKIs are under investigation in several malignancies, and preliminary data in BTC suggest intriguing signs of potential benefit in selected patient populations. Even though the results of this approach have not been always consistent in other malignancies, the analysis provides the rationale to further evaluate such a strategy in larger trials. Companion biological studies could contribute to identify promising predictive biomarkers of benefit or resistance, to be subsequently validated in other series exploring the role of ICIs in BTC.

In conclusion, the contributions to this Research Topic addressed different issues in the management of BTC. Through this Research Topic, we hope we have offered some fruitful suggestions for both practice and research to the readers of *Frontiers in Oncology*.
